# Validation and evaluation of diagnostic tests for *Schistosoma mansoni* in Brazil: The ValidaXisto study protocol

**DOI:** 10.1371/journal.pone.0350547

**Published:** 2026-06-02

**Authors:** Cristina Toscano Fonseca, Rosiane A. da Silva-Pereira, Roberta Lima Caldeira, Wilma Patrícia de Oliveira Santos Bernardes, Clarice Carvalho Alves, Taynãna César Simões, Wagnner José Nascimento Porto, Edward José Oliveira, on behalf of the ValidaXisto team

**Affiliations:** 1 Grupo de Pesquisa em Biologia e Imunologia de Doenças Infecciosas e Parasitárias, Instituto René Rachou, Fundação Oswaldo Cruz, Belo Horizonte, Minas Gerais, Brazil; 2 Instituto Nacional de Ciência e Tecnológica em Doenças Negligenciadas no contexto de uma só saúde no nordeste brasileiro _INCT-Negli-One, Universidade Federal Rural de Pernambuco, Recife, Pernambuco, Brazil; 3 Grupo de Pesquisa em Helmintologia e Malacologia Médica, Instituto René Rachou, Fundação Oswaldo Cruz, Belo Horizonte, Minas Gerais, Brazil; 4 Núcleo de estudo em Saúde Pública e Envelhecimento, Instituto René Rachou, Fundação Oswaldo Cruz, Belo Horizonte, Minas Gerais, Brazil; 5 Laboratório de Parasitologia, Instituto de Ciências Biológicas e da Saúde, Universidade Federal de Alagoas, Maceió, Alagoas, Brazil; 6 Grupo de Pesquisa em Genômica Funcional de Parasitos, Instituto René Rachou, Fundação Oswaldo Cruz, Belo Horizonte, Minas Gerais, Brazil; Institute of Tropical Medicine: Instituut voor Tropische Geneeskunde, BELGIUM

## Abstract

The persistence of schistosomiasis as a serious public health problem can be attributed, in part, to the lack of more accurate diagnostic techniques, particularly in areas with low prevalence and low parasitic burden. Thus, an effective diagnostic tool with broad applicability for detecting active infections in both high- and low-prevalence settings, as well as for accurately monitoring cure after therapeutic interventions, represent an instrument of utmost importance for controlling disease transmission. Therefore, we propose evaluating different commercial tests for schistosomiasis diagnosis identified through a review of all tests registered to be used in Brazil by the Brazilian regulatory agency (ANVISA) and those registered by other regulatory agencies, as well as prototypes of tests under development. In addition, we propose the development and evaluation of molecular diagnostic methodologies for schistosomiasis using different biological samples (stool, blood, and urine) from individuals living in an endemic area. The Kato-Katz technique, with 18 slides per stool sample, will be used as the reference test for definition of schistosomiasis cases. The performance of the evaluated techniques will be compared with respect to sensitivity, specificity, accuracy, positive and negative likelihood ratios, agreement with the reference test, cost, time, and ease of execution. The effectiveness and feasibility of these diagnostic tests will be assessed to recommend their incorporation into the schistosomiasis surveillance actions of the Ministry of Health within the Brazilian Unified Health System (SUS) in the short to medium term, considering the conditions faced by surveillance programs within Primary Health Care.

## Introduction

Intestinal schistosomiasis is a parasitic disease caused by trematodes of the genus *Schistosoma*, with *Schistosoma mansoni* Sambon, 1907 being the etiological agent of this disease in Brazil. The World Health Organization (WHO) estimates that schistosomiasis affects more than 240 million people worldwide and that over 700 million people live in areas at risk of infection [1]. The disease is directly associated with impoverished communities lacking access to safe water and adequate sanitation conditions [1]. In Brazil, schistosomiasis continues to be regarded as a serious public health problem [2]. It is currently present in 19 Brazilian states, being endemic in eight: Alagoas, Bahia, Pernambuco, Rio Grande do Norte, Paraíba, Sergipe, Espírito Santo, and Minas Gerais. Between 2009 and 2019, according to data from the Information System of the Schistosomiasis Control Program (SISPCE), the positivity rate for *S. mansoni* in endemic areas ranged from 5.20% in 2009 to 2.90% in 2018, with a total of 423,117 cases recorded [3].

Control measures for schistosomiasis have led to elimination or significant reductions in prevalence and infection intensity in many countries [4]. Consequently, there is a growing need for more sensitive diagnostic tests to determine the true prevalence of schistosomiasis, to monitor cases, and to certify elimination of transmission [5]. Currently, schistosomiasis cases are commonly defined by the observation of *S. mansoni* eggs through parasitological examination of slides prepared with human feces. The Kato-Katz technique [6,7] is the methodology recommended by WHO and the Brazilian Ministry of Health [8] due to its high analytical specificity, low cost, and relative simplicity of execution. However, the test’s performance may be affected by low parasite burden in the host, intermittent egg excretion, and uneven distribution of eggs within fecal samples [9], leading to underestimation of disease prevalence, especially in low-endemicity settings [10]. To minimize these limitations, researchers have recommended preparing 14–24 slides per fecal sample, instead of the two slides recommended by public health agencies in Brazil and globally. This approach allows a larger quantity of feces to be analyzed, increasing the probability of egg detection, and improving the reliability of results [11,12].

Serology represents a screening tool that may play an important role in the elimination process of schistosomiasis transmission [13]. The serological tests registered by the Brazilian Health Regulatory Agency (ANVISA) and commercially available in Brazil by April 2024 were manufactured in Germany by Euroimmun Medizinische Labordiagnostika AG and NovaTec Immundiagnostica GMBH. These account for nearly all serological tests performed by private laboratories in the country [14]. However, carefully designed and well-controlled studies are still required to validate the use of these and other tests in Brazil, particularly in endemic areas.

Additionally, antigen-detection tests may serve as useful confirmatory tools for seropositive individuals or as primary diagnostic methods. The possibility of using decentralized antigen detection tests makes them even more promising for epidemiological surveillance of the disease. Although literature data indicate excellent performance of circulating cathodic antigen (CCA) and circulating anodic antigen (CAA) detection tests for diagnosing active infection [15,16], the evaluation of a commercial point-of-care test for CCA detection (POC-CCA) in a multicentric study in Brazil revealed limitations in both sensitivity and specificity when applied to the Brazilian epidemiological context [13,17–19]. These shortcomings have been attributed to manufacturing issues, as evidenced by a study that identified flaws in some kit batches [20]. A new commercial test for CCA detection, POC-CCA3 is currently under development by EASE Dx Africa aiming to overcome the limitations observed with the POC-CCA.

Beyond antigen detection, molecular tests targeting parasite genetic material also hold great potential as confirmatory diagnostic tools due to their high sensitivity and specificity. Quantitative real-time PCR (qPCR) for parasite DNA detection in stool samples was standardized by IRR researchers [21] and evaluated by our group, showing high sensitivity and specificity [13]. However, its application requires complex laboratory infrastructure. Isothermal amplification techniques, as the Loop-mediated isothermal amplification (LAMP) and the Recombinase Polymerase Amplification or Recombinase-aided amplification (RPA/RRA) have emerged as simpler molecular alternatives, with the potential for use even in laboratories with limited infrastructure. Our studies demonstrate that LAMP shows high sensitivity and specificity (though lower than qPCR) when using DNA extracted from stool samples [13]. The challenge lies in reproducing these accuracy levels with less invasive biological samples, such as urine, and with samples already collected during screening, such as plasma.

Therefore, the Valida-Xisto project proposes laboratory validation of serological tests for schistosomiasis diagnosis, registered by ANVISA and other regulatory agencies and commercially available in Brazil, using serum samples from individuals living in an endemic area, classified as either schistosomiasis cases or non-cases. In addition, we will evaluate the performance of a new immunochromatographic test developed by Mondial Dx, and currently produced by EASE Dx Africa, the POC-CCA3 for detecting circulating cathodic antigens in urine samples from the same participants. Plasma, stool, and urine will also be used for evaluating LAMP and RAA-based assays for detecting cell-free DNA (cfDNA) in these samples. Results will be compared with those obtained from the qPCR stool assay already standardized by our group.

The performance of these diagnostic tests will be assessed in terms of sensitivity, specificity, accuracy, repeatability, reproducibility and reported according to Standards for the Reporting of Diagnostic Accuracy Studies (STARD) to generate evidence that may support their incorporation, in the short-to-medium term, into schistosomiasis surveillance programs conducted by the Brazilian Ministry of Health.

## Methods

### Study design

**Fig 1:**
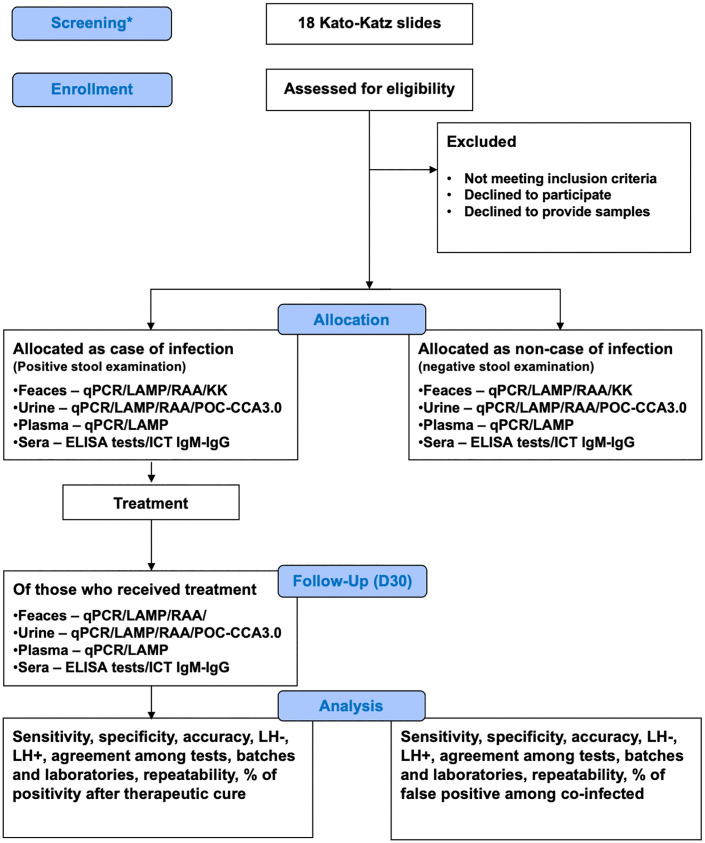
Study flow diagram. *performed by the SaneaXisto project team and by the municipal health department of Jaboticatubas.

This is a cross-sectional diagnostic accuracy study with a 30-day follow-up for post-treatment reassessment. The 30-day follow-up post treatment reassessment intend to assess the performance of the evaluated tests in determining cure. Individuals living in schistosomiasis-endemic areas from the state of Alagoas and Minas Gerais, Brazil will be evaluated using the Kato-Katz technique (18 slides prepared from a single stool sample from each participant) by the SaneaXisto project (CAAE: 71434823.4.1001.5091) or by the municipal health department of Jaboticatubas and other endemic municipalities in Alagoas. All individuals enrolled in the study after the screening performed by the municipal health department will have new samples collected and examined by Kato-Katz, performed technicians from the study team. This procedure aimed to ensure harmonization of the reference test procedures across all participants. the Those who do (cases) or do not (non-cases) present *S. mansoni* eggs in stool in the screening phase will be invited to participate in the study until sample size is achieved (Fig 1). Upon enrollment, each participant will provide one stool sample, two urine samples (collected in containers with and without preservatives), and two 5-mL blood samples (one collected in an EDTA tube to obtain plasma for molecular assays and one collected in a tube without anticoagulant to obtain serum), which will be used to perform the diagnostic tests evaluated in the study (Fig 1). Participants positive for *S. mansoni* who get treated with praziquantel will have stool, blood, and urine samples collected again 30 days after treatment (Fig 1).

### Eligibility criteria

Participants living in schistosomiasis-endemic areas, aged over 7 years, who screened positive or negative to the presence of *S. mansoni* eggs in feces will be invited to participate of this study. Those participants screened as non-infected who present *S. mansoni* eggs in a new stool sample collected after enrollment will be excluded. Participants screened as having active infections who report having received praziquantel treatment between screening and enrollment in our study will also be excluded. Pregnant or breastfeeding individuals and those who fail to provide any of the required biological samples will also be excluded from the study.

### Sample size calculation

For the sample size calculation, we used the lowest estimated sensitivity and specificity among the tests to be evaluated, according to data provided by the kit manufacturers and reviewed by Ramos and collaborators [14]. To determine the positive serum panel, we considered the sensitivity of the worst-performing test: ELISA IgG anti-*Schistosoma mansoni* (Euroimmun) – Sensitivity 73%. For the negative serum panel, we considered the specificity of the worst-performing test: IgM anti-*Schistosoma mansoni* (Euroimmun) – Specificity 62%. Type I error was set at 5% (α = 0.05; 95% CI) with a 10% half-width of the confidence interval. For the sample size (N), the following equation was used [22,23]: N = Z × Z [P(1 − P)]/(D × D),N = Z × Z[P(1 − P)]/(D × D), where: P = expected proportion (% Sens or Spec), D = margin of error and Z = 1.96 (for α = 0.05; 95% CI). Thus, the minimum sample would be 76 serum samples from positive individuals and 91 serum samples from negative individuals for schistosomiasis. However, because some tests have unknown accuracy estimates, this study aim to include 100 participants with active infection and 100 participants without active infection.

### Sample collection

Approximately 10 mL of blood will be collected using the Vacutainer® system into two 5-mL tubes: one containing EDTA anticoagulant and one without anticoagulant, both labeled with the participant ID. The tubes will be maintained at room temperature for up to three hours, centrifuged at 5,000 g for 10 minutes, and then the liquid fraction (serum and plasma) will be aliquoted into cryovials before freezing at −20 °C or −80 °C.

The urine, preferably the first morning urine, will be collected in two separate vials, one with preservative (10 mL) and one without preservative (10 mL). Between collection and delivery, samples will be kept refrigerated. Urine aliquots will be stored at −20 °C in the field and at −80 °C in the research center. Urine will be used for the POC-CCA 3 test and for cell-free DNA extraction.

When possible, the stool sample will be collected on the same day as the urine sample. After collecting the material may be kept at room temperature for up to two hours. If the interval between collection and delivery exceeds this period, it should be refrigerated for up to 24 hours. Aliquots of feces will be kept at -20^o^C in the field and then stored at –80^o^C in the research center.

### Diagnostic methodologies

#### Kato-Katz technique.

For parasitological diagnosis, stool samples will be processed by the Kato-Katz technique [7], preparing 18 slides from the same sample. The parasite load for each participant will be calculated as eggs per gram of feces (EPG). For quality control, 20% of the participants’ samples, randomly selected, will be sent to the Schistosomiasis Reference Laboratory at the René Rachou Institute – Fiocruz Minas. In case of discordant results, the reference laboratory result will be considered definitive.

#### Immunological tests.

Serum samples will be used to perform serological tests for schistosomiasis diagnosis using the following commercial kits: ELISA IgG anti-*Schistosoma mansoni* (Euroimmun), ELISA IgM anti-*Schistosoma mansoni* (Euroimmun), ELISA IgG anti-*Schistosoma mansoni* (NovaTec), ELISA IgM anti-*Schistosoma mansoni* (NovaTec), *Schistosoma mansoni* ELISA Kit (Bordier Affinity Products) and ICT IgG-IgM (LDBIO). Commercial tests will be performed according to the manufacturers` instructions. Urine samples will be tested by POC-CCA 3 test. The test will be performed according to the manufacturer’s instructions.

#### DNA extraction.

Urine and plasma samples will be used for cfDNA extraction using MagMAX™ Cell-Free DNA Isolation Kit (Thermo Fisher scientific) for LAMP and qPCR. The cfDNA extraction kit for the RAA test is being evaluated. This DNA will be used as a template for amplification by qPCR and LAMP (plasma and urine) and RAA (urine). Stool samples will be used for DNA extraction using QIAamp PowerFecal Pro DNA Kit (Qiagen) for qPCR and LAMP and QIAGEN DNEasy blood and tissue extraction kit and MPbio FastPrep bead-beat method for RAA. The extracted DNA will be stored at −80 °C until use in molecular tests.

#### Real-time qPCR.

The real-time qPCR assay will be performed as described by Siqueira et al., 2021 [21]. Genomic DNA samples will be evaluated by multiplex real-time PCR, targeting the *S. mansoni* repetitive genomic region (accession M61098), described by Hamburger and cols [24] and the human β-actin gene (*H. sapiens* ACTB), described by Musso et al. 1996 [25] as internal control. Reactions will be performed as previously described [21]. For each run, a positive control (mix plus DNA extracted from adult worms) and a non-template control (NTC) will be used. Amplification will be conducted on a ViiA 7 Real-Time PCR System (Thermo Fisher Scientific Inc., USA) using a universal cycling program with 45 cycles and an annealing temperature of 60 °C. DNA extraction and PCR microplate setup will be performed in separate rooms, in a laminar flow hood previously irradiated with ultraviolet light, using only sterile disposable products, including filter tips. Human stool DNA samples with Cq (quantification Cycle) below 42 for the target and below 44 for the internal control will be considered positive [21], for cfDNA, the threshold has not yet been determined. Commercial Kit -IBMP BioMol Esquistossomose (IBMP, Brazil), a qPCR KIT for *Schistosoma mansoni* detection will also be evaluated according to manufacturer’s instruction.

#### LAMP.

The primers described by Fernández-Soto et al. (2014) will be used [26]. The reaction protocol will be performed as previously described by Mesquita et al. (2022) [13]. The reaction will be incubated at 65°C for 50 minutes, followed by 80°C for 5 minutes to inactivate the enzyme. Amplification will be visualized using 2 µL SYBR Green I diluted 1:10 for color change (orange: negative; yellow: positive) by the naked eyes or under UV light (320 nm—non-fluorescent: negative; fluorescent: positive). For quality control and confirmation, 3 µL of the amplified product will be visualized on a 6% silver-stained polyacrylamide gel. Analytical sensitivity and specificity of the LAMP assay were previously determined by Mesquita et al. (2022) [13].

#### Recombinase-aided amplification (RAA).

RAA reactions will be performed in using the RAA Nucleic Acid Amplification Kit – Fluorescent Method (QT Biotech Co, Ltd) according to manufacturer’s instructions using primers and probes designed to *S. mansoni* target. cfDNA extract from urine will be used as a template. A positive (gDNA from adult worm) and a negative non-template (NTC) reaction will be performed in each run. Each reaction tubes will be mixed and incubated for 4 minutes in a 0.2-mL PCR tube under constant agitation and 42^o^C temperature. Fluorescence values will then be recorded every 20 seconds for a total of 16 minutes using a fluorometer.

### Analytical plan

Primary outcomes will be the sensitivity, specificity, accuracy, and positive and negative likelihood values of the tests. The Kato-Katz technique (18 slides prepared from one participants`s stool sample) will be used as reference tests. Participants with at least one *S. mansoni* egg detected in the feces will be considered infected (case). Participants characterized as non-infected must have negative results for *S. mansoni* eggs on all 18 slides examined (non-case). All index tests will be blinded evaluated.

Secondary outcomes will include the agreement index among tests, between different production batches, and between different operators for the commercial tests evaluated; the percentage of coinfection among false-positive individuals; and the percentage of positivity after therapeutic cure.

Summary statistics (means, percentiles, and measures of variability), agreement coefficients, and parametric and/or nonparametric hypothesis tests will be used depending on the distributions of collected and observed data [27]. Evaluation of diagnostic test accuracy estimates will initially be conducted using tests of homogeneity of proportions at a 5% significance level [28]. The R statistical software and the *Epipackage* will be used and [29,30]. The confidence interval for tests sensitivity and specificity and for positive and negative likelihood ratios will be calculated will be calculated according with Collett, 2002 [31] and Simel et al. (1991) [32], respectively.

Repeatability and reproducibility of commercial tests will be assessed. Repeatability will be evaluated using three different samples with different levels of reactivity for the immunological tests and different Cqs for the qPCR test. For each test, six aliquots of the same sample will be assayed on the same day. Reproducibility across batches (3 different batches), operators and laboratories (Imunological tests) or equipment (qPCR), and testing days (2 operators on 3 different days each) will also be assessed using a panel of 20 samples from infected individuals (case) and 20 samples from non-infected individuals (non-case). The coefficient of variation will be calculated to determine test repeatability and reproducibility.

### Data management plan

Data management will be carried out in accordance with Good Clinical Practices (GCP). All project members will take the GCP and good clinical laboratory Practices (GCLP) courses. Participants’ test results will be collected and/or entered source documents developed specifically for this study. The investigators will ensure that data entry, data review, and all required reports are complete, accurate, and performed in a timely manner, in accordance with any instructions provided. Project data will be entered into Research Electronic Data Capture (REDCap). The database will consist of eCRFs within REDCap. Personal identifying information will not be recorded in the eCRFs. Data entry into digital systems will be restricted to authorized study personnel. Electronic information will be stored on a server/system designated by the sponsor and in compliance with the Brazilian General Data Protection Law (LGPD). Source data and eCRFs, along with all ICF will be monitored by external members belonging to the Fiocruz`s clinical research platform Upon completion of the study data will be submitted for publication and deposited in a public repository.

### Safety considerations

This is not an interventional study, and the participants risks are mild and generally transient adverse effects related to blood sample collection and treatments. Venipuncture may cause pain, bruising, low blood pressure, dizziness or fainting. Blood collection will be performed by a qualified professional prepared to manage any complications. The use of praziquantel for schistosomiasis and albendazole for other helminth infections, although approved and considered safe, may cause transient symptoms such as abdominal pain, diarrhea, vomiting, nausea, dizziness, headache and vertigo. In addition, there is a residual risk of loss of confidentiality, which will be minimized by coding participants and keeping the link between identifiers and personal data stored only at the research site, with access restricted to the study team.

### Ethical considerations and declarations

The study was approved by the ethical committee of the René Rachou Institute (CAAE 79049624.0.0000.5091), which coordinated the study and by the ethical committee of the Federal University of Alagoas (CAAE 79049624.0.0000.5091) a coparticipant center. This study is being conducted in accordance with applicable regulations, including the Declaration of Helsinki and Brazilian National Health Council (CNS) Resolution No. 466/2012. The study also adheres to the standards established in ICH E6(R2) Good Clinical Practice (GCP) and the Good Clinical Practices: Document of the Americas.

### Study timeline

Participant screening and recruitment was initiated in January 2025 and is estimated to be completed in May 2026. Sample collection and processing was also initiated and are estimated to be completed in July 2026. Reference test is being performed as samples are collected and are estimated to be completed in September 2026. Index tests evaluation is estimated to be completed in December 2026. Data analyses are estimated to be initiated at March 2027 after the study database monitoring is completed. The study is estimated to be completed in April 2027.

### Laboratory and field personal training

All study team was trained in good clinical practices, in the study protocol and in the study operational procedures (SOP). The laboratory team was also trained in good clinical laboratory practices. Technicians involved in the manufacture of the Kato Katz slides and microscopists from both SaneaXisto and ValidaXisto involved in slides analysis were trained in the specific SOP.

## Discussion

In the absence of an effective vaccine against schistosomiasis and of an environmental intervention capable of sustainably interrupting transmission implemented, disease diagnosis arises as an important tool for controlling disease transmission [33,34]. An accurate diagnosis test enables the identification of infected individuals and its treatment. This study was designed to evaluate the diagnostic accuracy of different tests currently available or under development for schistosomiasis diagnosis, including commercial kits, prototypes, and molecular tests under laboratory development.

By investigating the performance of these tests in samples from individuals residing in an endemic area, including both cases and non-cases, this protocol aims to generate robust evidence to support decisions regarding the incorporation and implementation of these tools in the epidemiological surveillance of schistosomiasis in Brazil. It is important to highlight that the current epidemiological scenario in Brazil poses additional challenges to accurate diagnosis, as there is a need for a test with high sensitivity and specificity to detect the low-intensity infections, which are prevalent in Brazil [35].

Although the Kato–Katz method was adopted as the reference test, this study sought to minimize the inherent limitations of this technique by performing an extended reading of 18 slides prepared from a single stool sample per individual [11]. Nonetheless, given the intermittent egg excretion and the low infection intensity currently observed in Brazilian endemic areas, the possibility of false negatives among individuals classified as non-cases remains [36]. This potential misclassification in the control group may directly affect the estimated accuracy of the evaluated tests, particularly regarding specificity. In addition, the endemicity profile of the study area may influence the performance of the evaluated tests, especially serological assays, and must therefore be considered as a critical factor in the interpretation and extrapolation of the results. To minimize the impact of the epidemiological profile of endemic areas, participants will be recruited from different endemic settings in the states of Alagoas and Minas Gerais.

Additionally, this study aims to evaluate diagnostic performance of molecular tests in non-invasive (urine) and convenient (plasma) biological samples targeting cfDNA. This approach requires great care in the collection, preservation, and storage of the samples, as well as in the selection of the kits used for cfDNA extraction. To ensure sample quality, the group carried out standardization assays for collection, preservation, and storage, and the evaluation of different DNA extraction kits for qPCR and LAMP. The DNA extraction kits for performing RAA are still being evaluated by the group.

It is important to emphasize that the study design — with all assays performed in a central laboratory under direct supervision of the research team — does not constitute a field validation. The performance estimates reflect controlled conditions and technical expertise and may differ from what would be observed in decentralized programmatic settings. Thus, future multicenter implementation studies in health services are recommended, incorporating operational metrics (feasibility, time to result, cost, and acceptability) and comparisons with usual workflows. Despite the limitations outlined, this study represents an important step toward informing schistosomiasis epidemiological surveillance guidelines in Brazil.
